# Whom to Groom and for What? Patterns of Grooming in Female Barbary Macaques *(Macaca sylvanus)*

**DOI:** 10.1371/journal.pone.0117298

**Published:** 2015-02-10

**Authors:** Veronika Roubová, Martina Konečná, Petr Šmilauer, Bernard Wallner

**Affiliations:** 1 Department of Zoology, Faculty of Science, University of South Bohemia, České Budějovice, Czech Republic; 2 Department of Zoology, Faculty of Science, University of South Bohemia, České Budějovice, Czech Republic; 3 Department of Ecosystem Biology, Faculty of Science, University of South Bohemia, České Budějovice, Czech Republic; 4 Department of Anthropology, University of Vienna, Vienna, Austria; Institut Pluridisciplinaire Hubert Curien, FRANCE

## Abstract

Grooming is one of the most conspicuous social interactions among nonhuman primates. The selection of grooming partners can provide important clues about factors relevant for the distribution of grooming within a social group. We analyzed grooming behavior among 17 semi-free ranging female Barbary macaques (*Macaca sylvanus*). We tested whether grooming is related to kinship, rank and friendship. Furthermore, we tested whether grooming is reciprocated or exchanged for rank related benefits (i.e. lower aggression and increased tolerance whilst feeding). We found that in general grooming was reciprocally exchanged, directed up the hierarchy and at the same time affected by friendship and kinship. Grooming was more frequent among individuals with higher friendship values as well as amongst related individuals. We also divided our data set on the basis of rank difference and tested if different power asymmetries between individuals affected the tendency to exchange grooming for rank related benefits and grooming reciprocation. In support of our initial hypothesis our results show that the reciprocation of grooming was a significant predictor of grooming interactions between individuals of similar rank, but not between those individuals more distantly separated in the social hierarchy. However, we did not find any evidence for grooming being exchanged for rank related benefits in either data set. Our results, together with previously published studies, illustrate the behavioral flexibility of macaques. It is clear that multiple studies of the same species are necessary to gather the data required for the solid comparative studies needed to shed light on patterns of grooming behavior in primates.

## Introduction

Grooming behavior involves the careful inspection and subsequent cleaning of other individuals’ fur and may occupy up to 20% of the daily time budget in non-human primates [1]. Moreover grooming is not compromised in the face of other demands that may appear more important (e.g. increased foraging) [1], which suggests that it is of significant importance for the individuals involved. Unsurprisingly, grooming has been recognized as a fundamental aspect of primate sociality.

Traditionally grooming has been considered to be an altruistic behavior that is costly to the groomer (e.g. lost time and energy) and beneficial to the recipient (e.g. parasite removal or stress reduction) [1]. The time and energy devoted to grooming could otherwise be devoted to foraging, vigilance or finding a mate. Therefore individuals are expected to select a groomee who will be worth investing in, who may reciprocate the service and/or provide alternative benefits. Several theories have been suggested to explain the observed patterns of grooming in primates. For example, an explanation based on kin selection theory [2] would predict that there is selection for individuals to groom their own kin in preference to non-relatives in order to increase their own indirect fitness. Relatives benefit from reciprocal grooming exchange or the exchange of other benefits as well as non-relatives. However, the potential cost of non-reciprocation is lower to the groomer when the recipient is related. Although it has been shown that kinship has an effect on the grooming distribution within groups of primates, it is obviously not the only factor determining this behavior [3]. An alternative explanation is based on reciprocal altruism [4] and predicts that a groomer will favor a partner who is likely to reciprocate the investment. Reciprocal altruism is predicted to result in patterns of grooming reciprocation among individuals, with grooming being exchanged reciprocally. The concept of exchange has been broadened by biological market theory [5], which allows individuals to trade grooming not only reciprocally but also for other benefits. Some of these benefits can be provided mainly by high ranking individuals (e.g., tolerance around food or lower aggression) and thus exchange for these rank related benefits may explain the common pattern of grooming up the social hierarchy [1,6].

Strong positive social relationships (sometimes referred to as “friendship” or “high relationship quality” [7,8]) have been discussed as an important factor in explaining patterns of interactions in primate social groups [8–12]. Cords and Aureli [12] introduced a complex approach designed to measure social relationships. These authors defined three components relating to the quality of the relationship between individuals: value, security and compatibility. Only recently has this theoretical proposal been tested empirically, although it remains for such tests to be carried out using a standardized approach. In this study we adopted the concept of friendship defined as a way how to differentiate the strength of social interactions based on positive interactions [8]. Although the two terms (friendship and relationship quality) are related, and have sometimes been used interchangeably, friendship is defined more loosely and its measurement is usually based on one or a few behaviors [7,8].

Silk et al. [11] suggested that social bonds play a vital role in females’ lives, and the ability to establish and maintain strong social bonds (e.g., through grooming) may have important fitness consequences for baboon females. Despite recognizing the importance of grooming only a limited number of studies have tested the effect of social bonds on other behaviors empirically. One exception to this is the field of reconciliation, where a number of studies have reported a positive correlation between friendship or relationship quality and the probability of post conflict reconciliation (see e.g., [7,12,13]). However, it is obvious that primates living in social groups do not interact in the same way with all group-mates. Furthermore, the probability of a given behavioral interaction (e.g., grooming) occurring between two individuals is strongly influenced by the characteristics of each partner and the quality of their mutual relationship [8,11,13,14]. For example, individuals spend more time grooming their favorite partner in comparison to less favored ones (for review see [1]). It is also predicted that a generally high level of social tolerance in a particular group or species may decrease the need for subordinate females to achieve tolerant relationships via grooming-tolerance exchanges with dominant females [1]. As such the grooming partners chosen by females may be selected according to friendship rather than social rank. The tendency for individuals to select grooming partners with which they had good social relationships has been found in several macaque species (*Macaca assamensis* [13], *M*. *arctoides* [7]). This trend is expected to be more common in species with a more tolerant social style (see below) [15]. Barbary macaques are considered to be tolerant and as such we expect that friendship will play an important role in partner selection for grooming interactions.

Patterns of grooming interactions and their driving factors are not expected to be the same across different species of primates. Several characteristics of social hierarchy, particularly dominance steepness, vary among species and may have important consequences for grooming interactions [1]. Dominance steepness represents a measure of power difference between adjacently ranked individuals and it has been used to characterize species of macaques along a despotic-tolerant gradient [16]. In groups of despotic species (e.g. rhesus macaques) with steeper dominance hierarchies individuals are expected to exchange grooming for rank related benefits more often than individuals in groups of tolerant species (e.g., Barbary macaques) [1]. In tolerant species it is possible that the power differential among individuals is so low that grooming reciprocation among individuals will prevail over the exchange of other commodities. Interactions among individuals from despotic species should also be more dependent on rank and kinship than those of more tolerant species, where friendship among group mates may be more than or equally as important as rank and kinship [16]. Moreover, even within a single species, the exchange of grooming for rank related benefits is expected to be more frequent between individuals with higher rank difference than between individuals closer in rank, who will be more likely to groom reciprocally [1]. A number of problems make testing this prediction difficult, resulting in a paucity of relevant studies. Firstly, we still do not have relevant data for all species of interest, although the number of species on which data is available is slowly increasing. Second, for most species we have to rely on data from a single group. This is especially troubling for primates as this order is known for its wide behavioral flexibility, often related to ecological and social conditions [17]. These conditions may results in different levels of competition, which in turn influence the steepness of dominance hierarchies and therefore grooming patterns, similarly group size and adult sex ratio have also been shown to affect grooming patterns [17,18].

Macaques are a suitable group for testing the predictions of inter- and intra-species variation in grooming patterns. The number of species studied has been growing and for some species (e.g., Japanese macaques, *Macaca fuscata*) data from different groups and populations are available. This well-studied species is highly variable with regards to social behavior, and serves as a reminder that data from several groups per species is important for detailed comparative studies (Table 1 in [17,19]). Despite the importance of replication for behavioral studies, the data used in most meta-analyses [3,17,20,21] are derived from only one or two groups per species. This is even more conspicuous when considering socially tolerant species which are less well studied in comparison to their despotic relatives. Therefore, from a comparative perspective it is important not only when studying different species but also different populations within each species. Only a rich and diverse database of studies enables robust tests of the proposed hypotheses.

**Table 1:** List of behaviors and their definitions used for analyses.

Behaviors recorded using focal continuous sampling	
Approach	An animal comes into proximity of one or more individuals, stays in proximity for at least 5 sec. and the approach is not motivated by another obvious reason such as food.
Displacement	One animal in any way drives away another from some kind of resource (place, shadow, food, partner) and then stays in place, the displacing individual may or may not use the resource.
Groom	An animal grooms the hair of another; it watches the groomed spot on the other’s body. It may, use its fingers or mouth to pick up some particles. The identity of groomer and groomee as well as the duration of the grooming episode was recorded.
Aggression	This category included all aggressive interactions between given individuals e.g., silent threat, hand threat, chase, attack and bite.
Behaviors recorded using instantaneous sampling	
Contact	Two or more animals are touching with any part of the body. Not engaged in any other defined behavior such as an embrace or grooming etc.
Proximity	Two or more animals are within a stretched arm’s length of each other but are not in physical contact.
Co-provisioning	Two animals simultaneously use one food source. They may be in physical contact or proximity.

Grooming in Barbary macaques has been studied from various perspectives [22–25]. In this study we investigated the pattern of grooming in semi-free-ranging female Barbary macaques living in one social group. We investigated the main predictors of grooming interactions on the level of dyads, including: i) characteristics of the pair (kinship, relative rank, friendship), ii) previously proposed exchange commodities such as grooming received and, iii) potential rank-related benefits (lower aggression received and higher tolerance during co-feeding). Moreover, we tested whether grooming interactions among females close in rank showed a different pattern and relationship with the proposed predictors than grooming interactions among females more distant in rank. This study is the first to test the hypothesis that pair based characteristics and exchange commodities in Barbary macaques may have different effect on grooming interactions according to the difference in rank between grooming partners.

## Methods

### Ethical statement

This study was fully observational and non-invasive and adhered to the legal requirements of Gibraltar. Approval to conduct the study was granted by the Animal Care Appointee of the Gibraltar Ornithological and Natural History Society (GOHNS) (no permission IDs were given).

### Study subjects

The study was conducted in the Apes’ Den troop of Barbary macaques living in the Apes’ Den in the Upper Rock Natural Reserve, Gibraltar. This group is semi-free ranging, provisioned daily by the Gibraltar Ornithological and Natural History Society (GONHS), and visited by tourists. The study included all 17 adult females (age ≥ 3 years) present in the troop at the beginning of the study (age of females ranged from 3 to 26 years; mean 11.2 years, age data provided by GONHS). The troop also included six adult males and up to 15 juveniles and infants. Three new immigrant males (two sub-adult and one young adult male) joined the troop in the second part of the study. All adult subjects were individually recognized and well-habituated to the presence of human observers (for more details on study site and subjects see [26]). Maternal kin relationships were obtained from the GONHS database. The kinship data were included in the analyses as kin (mother-daughter and sister-sister dyads) and non-kin.

### Behavioral data collection

Behavioral observations were collected during two study periods that overlapped with two mating seasons: between November 2007 and February 2008 referred to hereafter as season 1 and between October 2008 and February 2009, referred to hereafter as season 2. Females were observed using two methods of data collection at the same time: focal continuous sampling (30min focal period) and focal instantaneous sampling [27] (focusing on the same individual at 2 min intervals). Behaviors were recorded according to an ethogram that consisted over 50 items, this ethogram was prepared on the basis of previous studies in non-human primates [28,29]. Behaviors analyzed in this study are listed in Table 1 together with the data collection methods. Data collection was distributed equally for each individual both throughout the day (from 8:00 to 18:00) as well as throughout the entire study. Females were observed once in a given day at most (and on average 2.1 (SD ±0.05) and 1.6 (SD ±0.08) times per week in season 1 and season 2 respectively). Behavioral observations were made by two observers (MK and VR) who were trained in data collection and the use of the behavioral ethogram in advance of data collection. The reliability of simultaneous observations of a given individual by the two observers reached 93% before the beginning of the data collection. For all of the following analyses only interactions among females were analyzed. For the grooming behavior the start and end time, direction of the interaction and identity of the social partner were recorded. The grooming act was considered terminated if it stopped for ≥20s.

### Dominance hierarchy

The social dominance hierarchy among 17 females was assessed on the basis of dyadic displacement interactions between pairs of observed individuals. Displacement interactions have been used to assess dominance hierarchy in wide range of species (elephants [30]; fowl [31]; macaques [32]) and are based on the clear observed acceptance of a subordinate position by the displaced individual (which is not always the case in aggressive interactions) [33]. The displacement interactions were entered into two sociometric matrices, separated by season. The linearity of the dominance hierarchy was assessed by the linearity index h′ in MatMan 1.1.4 (Noldus 2003) [34]. Subjects were subsequently ranked based on their normalized David’s score (NDS) [35,36] computed on the basis of the Dyadic Dominance Index corrected for chance (Dij) [37]. The NDS is a method of ranking individuals that also takes the relative strengths of the opponents into account and serves as a basis for computing the hierarchical steepness measured as the absolute values of regression slopes in plots between NDS and the order of individuals [35].

### Friendship measurement

Several measures of social relationship strength or friendship based on different behavioral characteristics and spatial proximity measures have been used in previous studies including grooming [11], approach interactions [13], mutual contact [7], or mutual contact and proximity [8]. Given that grooming was our behavior of interest we cannot use the measurement of friendship based on grooming data for our analysis. First we computed 4 different friendship measurements for each dyad based on four behaviors: time spent in body contact, time spent in proximity (within 2m), approaches and grooming. All 4 were positively correlated (see Table S5 Table and S6 Table). Body contact represents more intimate spatial relationship then proximity and is less time dependent on grooming behavior then approach (in other words an approach always has to occur before grooming can be performed but the two individuals do not have to be in body contact before and/or after grooming). We therefore chose to assess the friendship on the basis of the time females spent in mutual body contact (see Table 1 for definition) as it was also used in previous studies [7,8]. The friendship that individual A has with individual B was computed as the amount of time individual A spends in contact with individual B divided by the average amount of time individual A spends in contact with all other females. This resulted in an asymmetrical description of the relationship within a given dyad as the relationship that A has with B does not have to be equal to that of B to A. We favor this friendship assessment because it has been shown that asymmetry is an important predictor in social interactions [9]. The matrix of friendship values is provided in S8 Table and S9 Table.

### Data analysis

Linear mixed effect models (LMM) were used to test the effect of kinship, rank and friendship on grooming interactions. Grooming was represented by two measurements: grooming rate (the sum of grooming acts when A grooms B divided by the total time of observation of the two individuals) and grooming time (the sum of grooming time when A grooms B divided by the total time of observation of the two individuals). These two variables were positively correlated in our study (Spearman, season1 r = 0.89, p<0.001, season2 r = 0.83, p<0.001, N = 17), however some previous studies have shown different results for grooming rate and time, thus we computed models for both. The grooming data were log transformed to increase the homogeneity of their variances. The dataset for each model comprised two lines per dyad (i.e., A-B and B-A) and in total included 272 lines for each season. All the LMMs analyses were run in **R 3.0.2** (R Core Team, 2013) using the lme4 package [38]. When presenting model results, we show the estimated effect sizes (regression coefficient estimates) and their 95% confidence intervals. In this way, we can falsify null hypotheses (typically rejecting them at α = 0.05 when a confidence interval does not cover a zero value), and we also obtain biologically more interesting information about the size of each effect, finally we can also quantify the reliability of such an effect size estimate [39]. Given the fact that the response variables were log-transformed, we can quantitatively interpret the estimated effect sizes (b_i_) by saying that the expected response variable value increases *exp(b*_*i*_ *)*-times (if the resulting value is < 1, then the change represents decrease) when the predictor value increases by one unit. In the case of categorical predictors (which all had only two states in our data set), *exp(b*_*i*_ *)* shows how many times larger the mean response value for the particular predictor state is than the mean of observations for the other (reference) state. This interpretation using the exponential function can also be applied to the end points of the estimated confidence intervals.

The models tested the effects of the following variables on initiated grooming rate or grooming time. **Rank—**represented the dominance relationship of the groomer relative to the groomee coded as two states: dominant and subordinate. **Rank distance**—represented by the absolute value of the rank distance between the groomer and groomee (based on the David`s score). **Kinship—**represented the maternal kin relationship between the groomer and groomee coded as two states: kin and non-kin. **Friendship—**measured as the time spent in contact by a given pair of individuals relative to average time spent in contact with other group members, entered as a continuous variable. **Co-provisioning—**measured as the percentage of time spent by individuals in a given dyad using one food source. **Grooming received—**grooming rate or time received by the groomer from a given groomee. **Aggression received**—rate of aggression received by a groomer from a given groomee. **Season—**coded as two states: season 1 and season 2, corresponding to the two study periods. **Age difference**—the absolute value of age difference between the groomer and the groomee. This last variable was added to control for dyad similarity based on age. The identities of the groomer and groomee were used as crossed random factors in the models.

In order to assess the differences in grooming patterns of individuals closer in rank compared to those of individuals that were distant in rank, we divided our data into two subsets based on rank distance: a) a dataset with grooming interactions among females who have higher rank distance than the total sample average rank distance and b) a dataset with grooming interaction among females who have smaller than average rank distance. Again we tested the effect of the same variables on grooming rate and grooming time. This yielded four additional models. For each model we checked homoscedasticity and distribution of residuals using regression diagnostic plots. The significance of all our models was assessed by comparison with corresponding null models.

## Results

### Grooming behavior

We collected a mean of 15.5 (±0.27 SD) hours of focal observation data per female in season 1 and 13.6 (±0.65 SD) hours per female in season 2. We recorded 1,362 grooming interactions among females (842 in season 1 and 520 in season 2). Females were involved in an average of 0.17 (±0.48 SD) grooming interactions per hour and for an average of 0.79 (±1.74 SD) minutes per hour. The number of grooming interactions per female ranged from 69 to 298 with an average of 160.2 interactions. Data provided in S1, S2, S3 and S4 Tables.

### Dominance hierarchy

The resulting dominance hierarchies were based on 495 (with 21 unknown dyads) and 395 (with 35 unknown dyads) interactions in season 1 and season 2 respectively. The dominance hierarchies were significantly linear (season 1: h' = 0.78, p < 0.001; season 2: h' = 0.63, p < 0.001) and the direction of interactions was highly consistent with the resulting rank order (directional consistency index [40] in season 1 DCI = 0.97; in season 2 DCI = 0.99). No rank changes were identified within each season, but there were several changes in rank order of particular individuals (by up to three positions) between the two seasons. The values of the hierarchical steepness gradient were 0.50 in season 1 and 0.40 in season 2. Both linearity and steepness values are comparable with a previous study of a different group of Barbary macaques; h' = 0.60, steepness = 0.48 [22].

### Grooming pattern (results of LMMs)

**Grooming distribution among all females.** Grooming rates were significantly related to kinship, relative rank, friendship, grooming received and aggression received (Table 2).

**Table 2:** Results of LMMs testing grooming rate and grooming time and their relationship to kinship, rank distance, relative rank, friendship, grooming received, co-provisioning, aggression received and season.

	Grooming rate				Grooming time			
Fixed effects	Estimate	SE	95% confidence interval		Estimate	SE	95% confidence interval	
Age difference	0.002	0.014	-0.026	0.030	0.004	0.019	-0.034	0.040
Kinship (no)	**-0.608**	**0.306**	**-1.222**	**-0.012**	-0.645	0.402	-1.451	0.140
Rank distance	-0.073	0.048	-0.167	0.026	-0.102	0.064	-0.226	0.028
Relative rank (s)	**0.587**	**0.214**	**0.148**	**1.045**	**0.929**	**0.292**	**0.330**	**1.554**
Friendship	**0.405**	**0.045**	**0.317**	**0.493**	**0.531**	**0.056**	**0.421**	**0.640**
Groom received	**1.229**	**0.038**	**0.496**	**1.959**	0.159	0.115	-0.063	0.383
Co-provisioning	0.140	0.141	-0.134	0.415	0.213	0.187	-0.149	0.577
Aggression received	**2.156**	**0.488**	**1.210**	**3.117**	**2.775**	**0.644**	**1.520**	**4.041**
Season (2)	**-0.363**	**0.152**	**-0.658**	**-0.063**	**-0.686**	**0.203**	**-1.079**	**-0.289**

Variables with values in bold had significant effect on grooming rate or grooming time based on CI.

Females groomed their kin more often than non-kin. The model predicted that in the absence of maternal kinship between the grooming partners the grooming rate is 1% to 71% lower when compared to grooming among maternal kin dyads. Females directed grooming up the hierarchy (i.e., most often the groomers were of lower dominance rank than groomees). The model predicted that when a groomer is subordinate to a groomee the rate of grooming is 16% to 184% higher than when the groomer is dominant to the groomee. Friendship was also a significant predictor of grooming and females groomed their higher quality partners more often than low quality social partners. The model predicted that an increase of 1 in the friendship value will lead to an increase in grooming rate of 37% to 64%.

The results also show that females exchange grooming for grooming with their partners as they more often groom females who groomed them back. The model predicted that an increase of 0.1 in grooming rate received is related to an increase of 5% to 22% in grooming rate initiated. Aggression received was also related to grooming rate although, contradictory to our hypotheses, this was in a positive direction, in other words those groomers who initiated more grooming received more aggression from that given partner. The model predicted that an increase of 0.1 in aggression is related to an increase from 13% up to 37% in grooming rate.

The season had also a significant effect on grooming, with females grooming less often during the second season. Neither relative rank distance among grooming partners nor time spent co-provisioning were related to grooming distribution in this or any of the following models.

The results were slightly different for the model based on grooming time (Table 2). Kinship was not a significant predictor and females did not groom their relatives for a longer time than their non-relatives. The amount of grooming received was also not a significant predictor in this model suggesting that the exchange of grooming among females is mainly based on frequency and not time. Relative rank, friendship and aggression received were significant predictors of the grooming time pattern. Thus females groom longer a) partners dominant to themselves, b) partners with which they have a better relationship and c) partners who target them with aggression.

**Comparison of grooming distribution among females close in rank and distant in rank.** Results of the LMMs based on the two separate data sets showed that grooming received was a significant predictor in grooming among females close in rank but not in grooming among females distant in rank. Females close in rank thus exchanged grooming reciprocally and females distant in rank did not (Table 3, Table 4). This was the case for grooming rate models but not for grooming time models (Table 3, Table 4). Moreover the aggression received by an individual was related to grooming distribution (both rate and time) among females close in rank but not among females distant in rank. Kinship was a significant predictor in grooming interactions among females distant in rank but not among females close in rank, although the direction of the relationship was similar. Relative rank was also a significant predictor in most of the models, thus females close as well distant in rank usually groomed females dominant to themselves more often and for longer periods of time. Finally friendship was also a significant predictor of grooming rate and grooming time in both datasets. Thus females close as well as distant in rank groomed more often and for longer time their friends. Our results thus suggest that grooming interactions among females close in rank and females distant in rank are not always affected by our predictor variables in the same way.

**Table 3:** Results of LMMs testing grooming rate and grooming time and their relationship to kinship, rank distance, relative rank, friendship, grooming received, co-provisioning, aggression received and season among females with low rank distance.

	Grooming rate				Grooming time			
Fixed effects	Estimate	SE	95% confidence interval		Estimate	SE	95% confidence interval	
Age difference	-0.002	0.020	-0.040	0.035	-0.003	0.025	-0.052	0.046
Kinship (no)	-0.446	0.350	-1.159	0.227	-0.532	0.452	-1.453	0.343
Rank distance	-0.031	0.146	-0.318	0.252	-0.042	0.188	-0.413	0.322
Relative rank (s)	0.423	0.243	-0.043	0.892	**0.766**	**0.317**	**0.159**	**1.381**
Friendship	**0.397**	**0.055**	**0.291**	**0.503**	**0.503**	**0.066**	**0.374**	**0.631**
Groom received	**1.243**	**0.417**	**0.429**	**2.052**	0.211	0.136	-0.052	0.478
Co-provisioning	0.094	0.163	-0.220	0.408	0.158	0.212	-0.253	0.567
Aggression received	**2.283**	**0.576**	**1.161**	**3.450**	**2.888**	**0.744**	**1.426**	**4.410**
Season (2)	-0.233	0.219	-0.655	0.194	-0.504	0.286	-1.058	0.053

Variables with values in bold had significant effect on grooming rate or grooming time based on CI.

**Table 4:** Results of LMMs testing grooming rate and grooming time and their relationship to kinship, rank distance, relative rank, friendship, grooming received, co-provisioning, aggression received and season among females with high rank distance.

	Grooming rate				Grooming time			
Fixed effects	Estimate	SE	95% confidence interval		Estimate	SE	95% confidence interval	
Age difference	-0.023	0.018	-0.058	0.013	-0.023	0.026	-0.072	0.027
Kinship (no)	**-2.105**	**0.790**	**-3.614**	**-0.571**	**-2.824**	**1.002**	**-4.743**	**-0.885**
Rank distance	-0.067	0.096	-0.251	0.117	-0.136	0.136	-0.394	0.122
Relative rank (s)	**1.485**	**0.352**	**0.807**	**2.155**	**2.097**	**0.519**	**1.107**	**3.077**
Friendship	**0.359**	**0.092**	**0.183**	**0.543**	**0.549**	**0.118**	**0.314**	**0.781**
Groom received	3.567	1.864	-0.049	7.159	0.099	0.233	-0.353	0.546
Co-provisioning	0.544	0.334	-0.106	1.186	0.819	0.443	-0.045	1.668
Aggression received	1.800	1.093	-0.290	3.921	2.342	1.498	-0.516	5.309
Season (2)	**-0.454**	**0.212**	**-0.865**	**-0.044**	**-0.858**	**0.289**	**-1.416**	**-0.301**

Variables with values in bold had significant effect on grooming rate or grooming time based on CI.

## Discussion

Our study determines the effects of three main factors on grooming interactions in female Barbary macaques, namely kinship, relative rank, and friendship. It also shows that the main reward for grooming is reciprocation, but no evidence was found for exchange of rank-related benefits (i.e. lower aggression and higher tolerance while feeding). In addition to these general patterns, we detected differences between pairs of individuals that were particularly close or distant in rank.

Of the factors studied here the effect of kinship and rank on grooming behavior has already been demonstrated (for reviews see [3,10,20,21]). However, the significance of kinship and rank in respect to other factors and social structure is not clear. For example, in their recent meta-analysis, Schino & Aureli [3] conclude that although kinship plays some role in grooming interactions among primates its importance is much lower than previously thought, particularly in relation to grooming reciprocation. Our data are compatible with this conclusion: the effect sizes suggested that although kinship is a significant predictor of grooming in some cases it was not an overriding factor in our models. A more complex scenario for rank-dependent effect has been suggested by Thierry [15]. This scenario suggests that the effect of dominance rank and kinship on social interactions should be of limited importance, perhaps negligible, in species with a tolerant social style (e.g. Sulawesi macaques), compared to those with a more despotic style (e.g. rhesus macaques). However, our results, as well as several previous studies, demonstrate that the effects of kinship (*M*. *sylvanus*, [22]) and/or rank (*M*. *thibetana*, [41]) is nevertheless recognizable and significant even in tolerant macaque species. This suggests that the behavioral patterns along the tolerance-despotism species range should indeed be seen as a continuum with inter-species overlaps rather than entirely distinct modes [15].

The third factor, friendship, was previously demonstrated to influence positive social interactions e.g. reconciliation [13] and this was confirmed by our analyses. Under this scheme females groom other females with whom they had a better relationship more frequently and for longer time periods. Although well-known and acknowledged, this factor has rarely been tested in one model together with the other factors, such as kinship and rank, to show its effect on grooming patterns. Our design proves that even after including the effect of rank and kinship, friendship remains a significant behavioral factor affecting the grooming distribution.

### Absence of rank related benefits

As mentioned above the significant relationship between grooming and rank was not surprising. The tendency of females to prefer a dominant grooming partner is usually explained by social market theory, where low ranking females have more to gain from high ranking individuals than vice versa [1,6]. As has been previously proposed and tested, subordinate females may exchange grooming for agonistic support from dominant individuals [21], their tolerance [1], or for access to desirable or scarce resources [42]. We did not find any potentially rank related benefits included in our models (decreased aggression received and higher tolerance during co-provisioning) to be related to grooming patterns. While slightly surprising, these results correspond to several studies reporting a lower tendency to exchange grooming for rank related benefits in other macaque species [42–45]. A possible theoretical background for such an observation may be provided by the prediction of Henzi and Barret [1]. They proposed that the pattern of grooming interactions is affected by the power differential between individuals, where a lower steepness of dominance hierarchy may lead to a lower demand for rank related benefits. In such a case, grooming would be exchanged for grooming, rather than for any other benefits. Based on their recent study of Assamesse macaques (*Macaca assamensis*), Macdonald and colleagues [45] also suggested that in the absence of competition there is no need to exchange rank related benefits among females. However, this explanation does not seem to fit our observations. The group under study was provisioned by local management with food in a single small patch that could be monopolized by several individuals. We thus expect the motivation of subordinates to exchange their grooming for tolerance while using this food resource to be high. Considering the condition of our study group, (i.e., high levels of stress due to tourism and unavoidable contact with human visitors), stress factors may provide an alternative explanation for the lack of exchange for rank related benefits. Balasubramaniam et al. [46] showed that stressful conditions may lead to a more intensive demand for grooming per se than to agonistic support or other potential rank related benefits. This view is based on the perception of grooming as a stress-reducing and relaxing mechanism [47,48]. We therefore suppose that stress factors (including the clumped provisioning of food) may have led to a higher demand for grooming interactions as a positive stress-reducing behavior.

Environmental conditions together with the more tolerant social system of Barbary macaques might explain the absence of exchange for potential rank related benefits. If this was the case we would also expect infrequent grooming up the hierarchy, but this remains present among females in our focal group. Moreover, exchange for rank related benefits has recently been documented in another study of Barbary macaques [22], although surprisingly this study did not find an effect of rank on grooming interactions. One possible explanation is that there are other rank related benefits that were not included in our model. One of such benefit is agonistic support. The incidence of agonistic support was very rare in our study group and thus impossible to analyze (only 29 instances in two seasons, and 52% of them were among the members of the largest matriline). However the significant relationship between grooming and agonistic support in general has been hard to find in previous studies [1] and as concluded by Shino [21] the relationship is rather weak, and given publication bias, probably overestimated.

We suggest that another relevant rank related benefit may be access to mates. Although competition among females for mates has been usually overlooked [49] it can represent a potentially important factor in a) Barbary macaques in general, as they breed seasonally and have partly synchronized ovulatory cycles [50], and in b) our study group in particular where adult sex ratio was skewed (1M:2.8F). Thus females may compete for access to males to gain mates. This can be motivated by the possibility of sperm depletion or by limitation of future resource competition, both factors previously reported for primates (reviewed in [49]). However, tolerance during mating was not tested in our models. Further studies comparing data from mating and non-mating seasons in the same group may shed more light on the role of mating competition on grooming interactions among females, and help to investigate the role of tolerance while mating as another potential rank related benefit for females.

### Relationship between aggression and grooming

An interesting and contradictory result of our study is the positive relationship between aggression received and grooming given. While lower aggression has been considered as one of the potential benefits to be gained via grooming interactions [51], in our study group females received more aggression from females whom they groomed more. For Barbary macaques this phenomenon is not atypical since it has been recently reported elsewhere [22,25]. The explanations suggested by these studies include grooming being used to appease aggressive individuals and its use in post-conflict reconciliation. Such situations might represent an opportunity for a former aggressor to coerce a victim by demanding grooming, with subordinates being more prone to succumb to such behavior when the power difference is high [25]. However our data did not provide much support for either of these views. In our focal group aggression was positively related to grooming only in dyads close in rank, but not in the dyads distant in rank. This observation is difficult to reconcile with the suggestion that higher rank distance should actually result in a situation where subordinates are highly motivated to appease or succumb to coercion, a pattern contradictory to our observations. It has also been suggested that the levels of aggression will be even higher without grooming and that individuals do in fact exchange grooming for reduced aggression [22]. However, this idea can only be tested using detailed behavioral time-sequence analysis or an experimental setup. Moreover our result showing that grooming is generally reciprocated also, in our opinion, does not fit with these explanations, e.g., if there is a motivation on the side of the aggressor to coerce a subordinate individual via aggression to grooming, we would not expect that he/she would also reciprocally groom the former victim. While these causal relationships are difficult to test we suggest an alternative and simpler hypothesis. We suppose that the higher incidence of grooming and aggression might be explained simply by the spatial and temporal associations of individuals, which determines the probability of interactions among individuals. In other words, this conclusion is based on the observation that individuals spending more time together grooming each other also have a higher probability of getting into a conflict. Furthermore, spending more time in close association is more typical for individuals close in rank compared to those distant in rank. Thus our results for the two data sets fit this explanation well.

### Grooming rate vs. grooming time

In our data we found several differences in models based on grooming rate compared to those based on grooming time. These differences suggest that individuals reciprocate grooming more on the basis of rate rather than time. In other words, individuals in a given dyad groom each other at the same rate but not necessarily for the same duration of time. This finding is unfortunately difficult to compare to other studies, as they usually use either time or rate as an exclusive parameter in their analyses (but see [41]) or do not discuss the differences. We may hypothesize that individuals can control or track the rate of interactions (make decisions on whether to approach a given individual or not) better than the duration of interaction. Moreover, the duration can also be influenced by other factors such as the decision by the groomee or actions of other individuals (e.g., fights, alarm calls etc.). We also assume that tracking time investment is more cognitively demanding then keeping track of frequency. However, regardless of the real causes for these differences, the usage of either frequencies or time of grooming may be another source of variation between studies, and measurements should therefore be well defined in such behavioral studies.

Finally it should be noted that statistical analysis of grooming data involves several limitations and potential issues of the reliability of the fitted models. First, the grooming interactions are not evenly distributed across all group members and thus the resulting dataset includes many zeroes. This is a natural consequence of the fact that individuals groom others selectively and thus some dyads groom each other more often than others and some do not engage in grooming at all. Second, some combinations of predictors included in our model are rare or even non-existent e.g., females distant in rank are more often unrelated than related. This again is an inevitable consequence of the nepotistic hierarchies and matriline system of macaques. These data characteristics may limit the power and possibly even the reliability of the tests but are also impossible to avoid.

## Conclusions

Our study tested the hypothesis that grooming is exchanged for different commodities in relation to rank difference among tolerant Barbary macaque females. We found that individuals in general, and specifically those close in rank, mainly exchange grooming reciprocally. Our results thus support the hypothesis that in tolerant species reciprocal grooming will prevail over exchange of grooming for rank related benefits. However, females still directed grooming up the hierarchy and thus further studies are needed to reveal if other rank-related benefits can explain this pattern. We also suggest that the relationship between aggression and grooming is more complex than previously thought. The prediction that grooming serves to reduce the aggression received from ones grooming partner has not been supported in Barbary macaques.

We thank Vaclav Hypsa for the encouragement and helpful comments made during writing of the manuscript, Stanislav Lhota for useful comments on the previous version of the manuscript and Simon T. Segar for language corrections. We also thank three reviewers for their detailed comments that significantly improved the manuscript.

## Supporting Information

S1 Table Grooming rate (per hour of observation) in season 1. (XLS)

S2 Table Grooming rate (per hour of observation) in season 2. (XLS)

S3 Table Grooming time (minutes per hour of observation) in season 1. (XLS)

S4 Table Grooming time (minutes per hour of observation) in season 2. (XLS)

S5 Table Correlations among friendship measurements based on contact, proximity, approach and grooming (season 1). (XLS)

S6 Table Correlations among friendship measurements based on contact, proximity, approach and grooming (season 2). (XLS)

S7 Table Friendship matrix based on time spent in contact (data season 1). (XLS)

S8 Table Friendship matrix based on time spent in contact (data season 2). (XLS)
